# Supplementary material for: Genome-wide association study and population structure analysis of seed-bound amino acids and total protein in watermelon
Source: PeerJ. 2021 Oct 19;9:e12343. doi: 10.7717/peerj.12343 (PMC8533027; doi:10.7717/peerj.12343)
Supplement: Supplemental Information 3 — The levels not connected by the same lower case (continents) and upper case (species) letters are significantly different (Student t-test; p = 0.05) [file peerj-09-12343-s003.pdf]

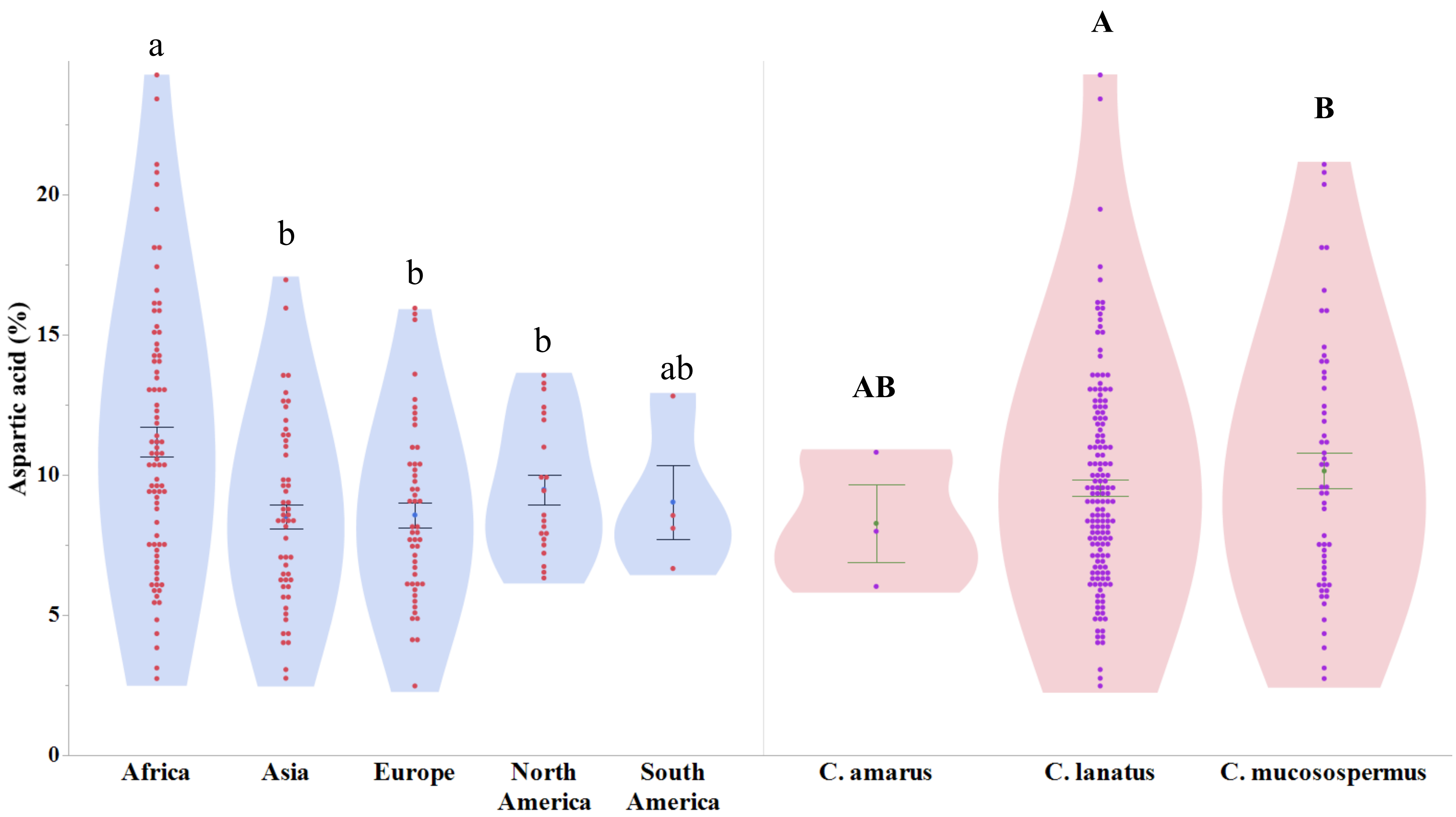

Supplemental Figure S3. Contour graphs showing variation in the percent aspartic acid content in watermelon accessions across geographies and species. The levels not connected by the same lower case (continents) and upper case (species) letters are significantly different (Student t-test; p=0.05)
