# Supplementary material for: Genome-wide association study and population structure analysis of seed-bound amino acids and total protein in watermelon
Source: PeerJ. 2021 Oct 19;9:e12343. doi: 10.7717/peerj.12343 (PMC8533027; doi:10.7717/peerj.12343)
Supplement: Supplemental Information 6 — Bi-plot for the first two components (PC) is shown for all seed-bound free amino acids in watermelon accessions (A) across continents (B) and species (C) [file peerj-09-12343-s006.pdf]

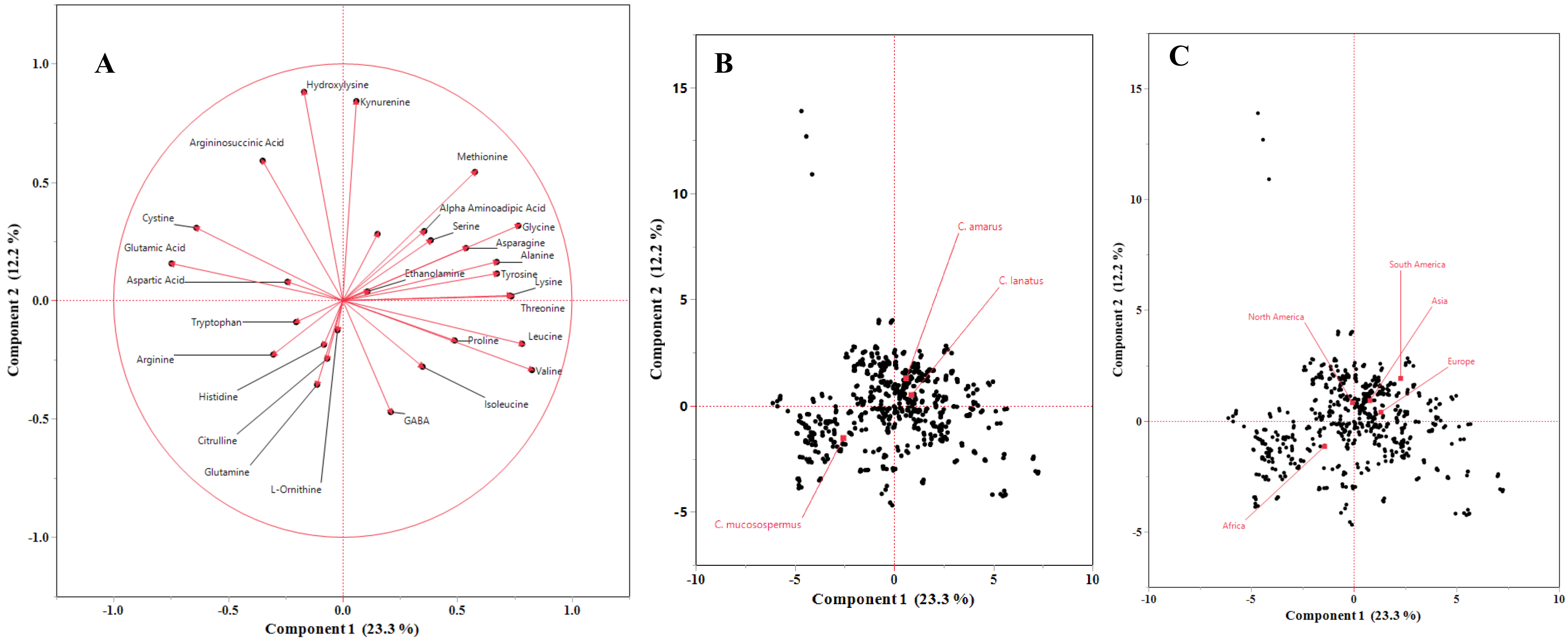

Supplemental Figure S6. Principal component analysis (PCA) of seed-bound amino acids. Bi-plot for the first two components (PC) is shown for all seed-bound free amino acids in watermelon accessions (A) across continents (B) and species (C)
