# Supplementary material for: Genome-wide association study and population structure analysis of seed-bound amino acids and total protein in watermelon
Source: PeerJ. 2021 Oct 19;9:e12343. doi: 10.7717/peerj.12343 (PMC8533027; doi:10.7717/peerj.12343)
Supplement: Supplemental Information 7 — The plots were drawn for expected vs. observed −log10 (p-values) for all amino acids [file peerj-09-12343-s007.pdf]

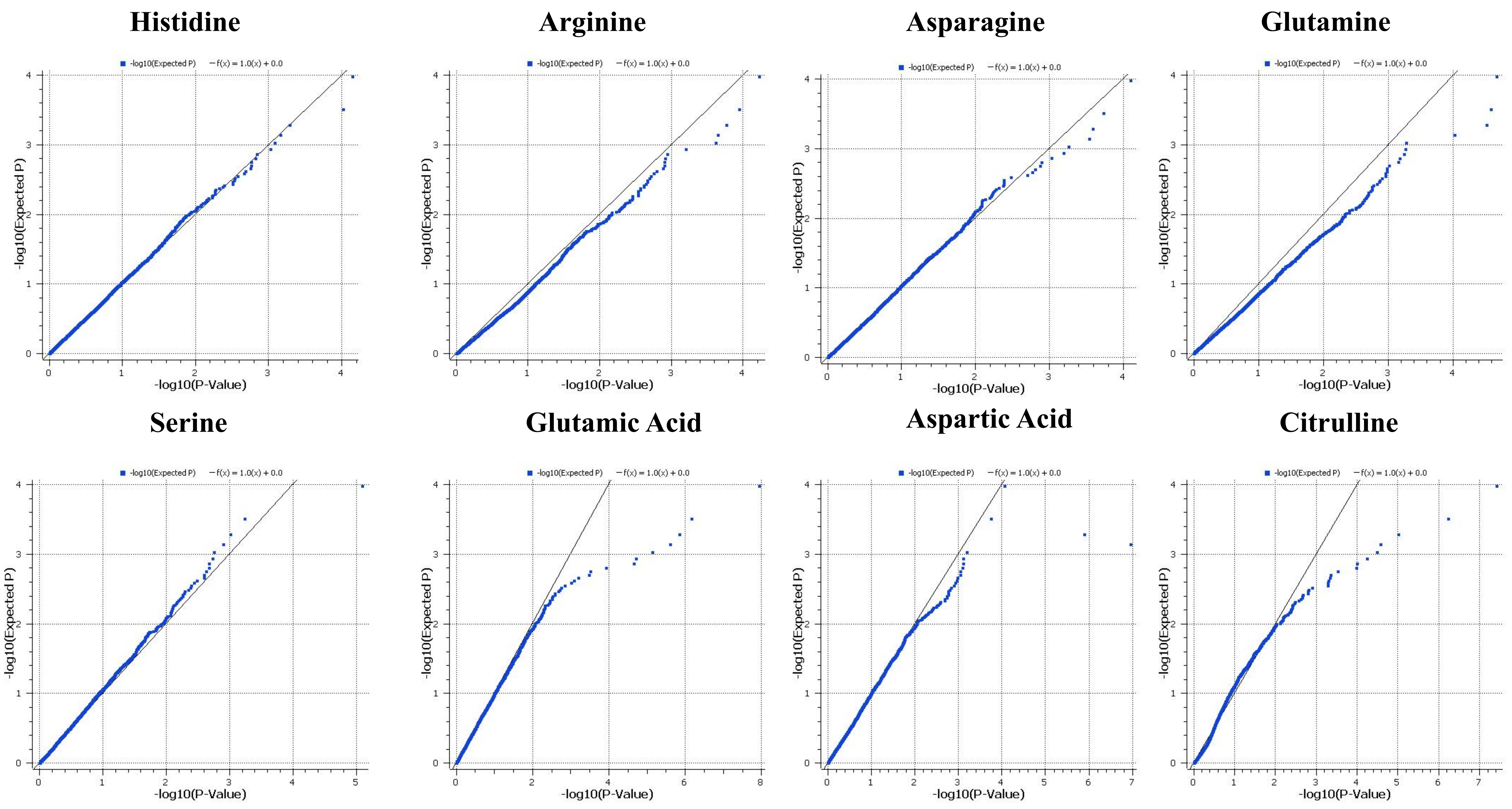
Histidine
Arginine
Asparagine
Glutamine
Serine
Glutamic Acid
Aspartic Acid
Citrulline
-log10(Expected P)
f(x) = 1.0(x) + 0.0
-log10(P-Value)

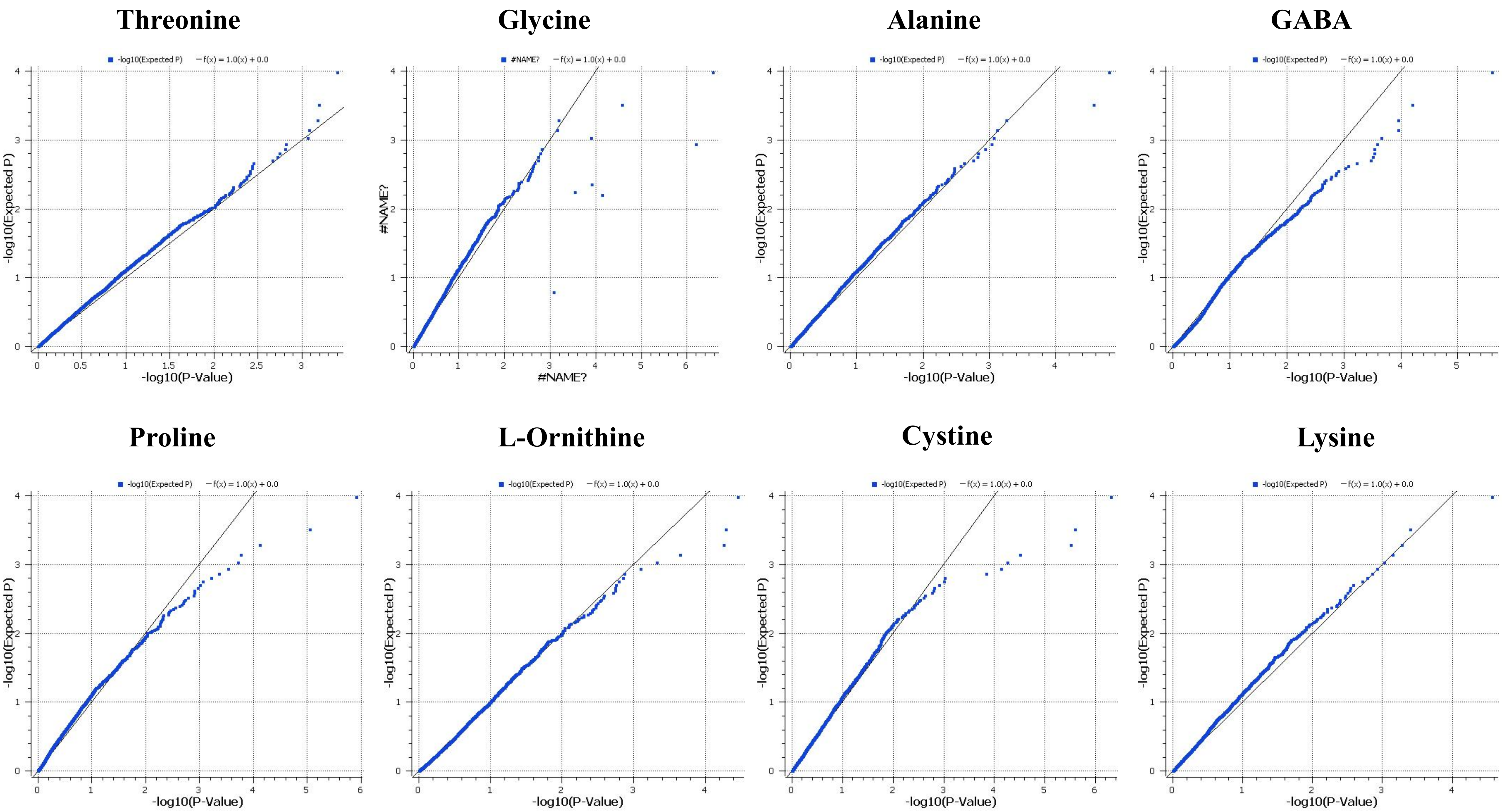

Threonine
-log10(Expected P)
f(x) = 1.0(x) + 0.0
-log10(P-Value)
Glycine
#NAME?
f(x) = 1.0(x) + 0.0
#NAME?
#NAME?
Alanine
-log10(Expected P)
f(x) = 1.0(x) + 0.0
-log10(P-Value)
GABA
-log10(Expected P)
f(x) = 1.0(x) + 0.0
-log10(P-Value)
Proline
-log10(Expected P)
f(x) = 1.0(x) + 0.0
-log10(P-Value)
L-Ornithine
-log10(Expected P)
f(x) = 1.0(x) + 0.0
-log10(P-Value)
Cystine
-log10(Expected P)
f(x) = 1.0(x) + 0.0
-log10(P-Value)
Lysine
-log10(Expected P)
f(x) = 1.0(x) + 0.0
-log10(P-Value)

Tyrosine

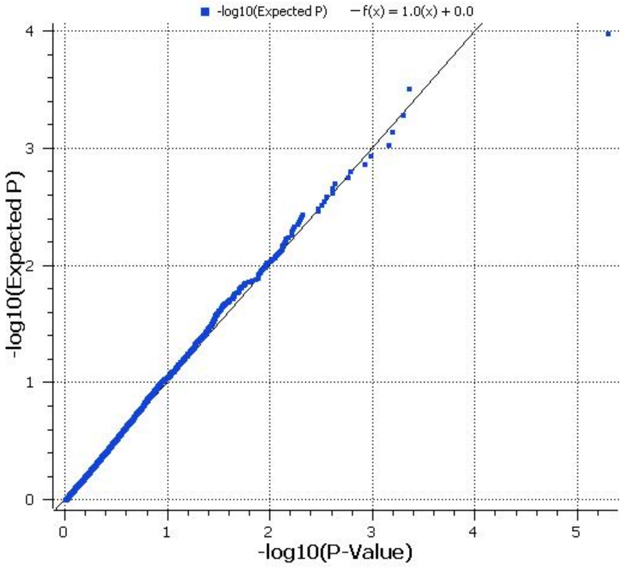

Methionine

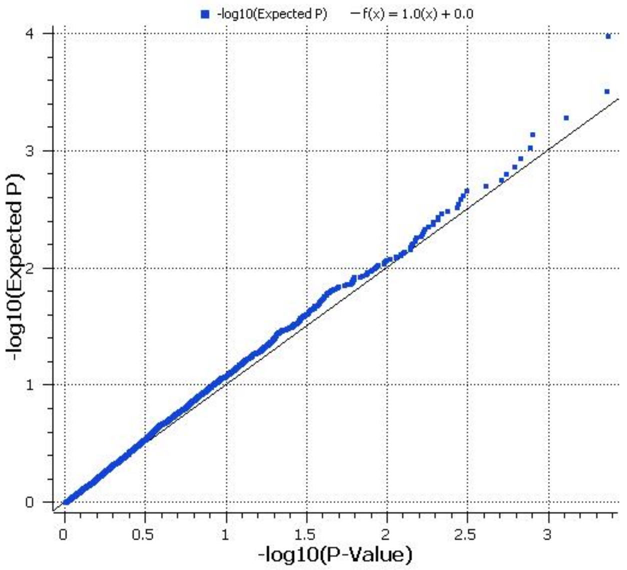

Valine

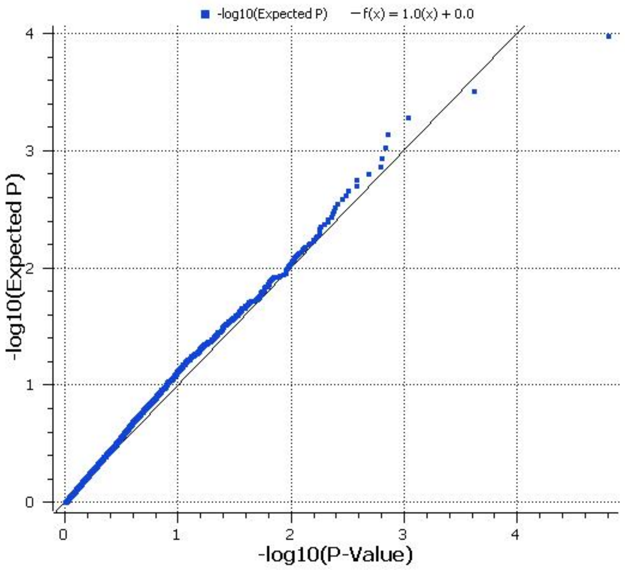

Isoleucine

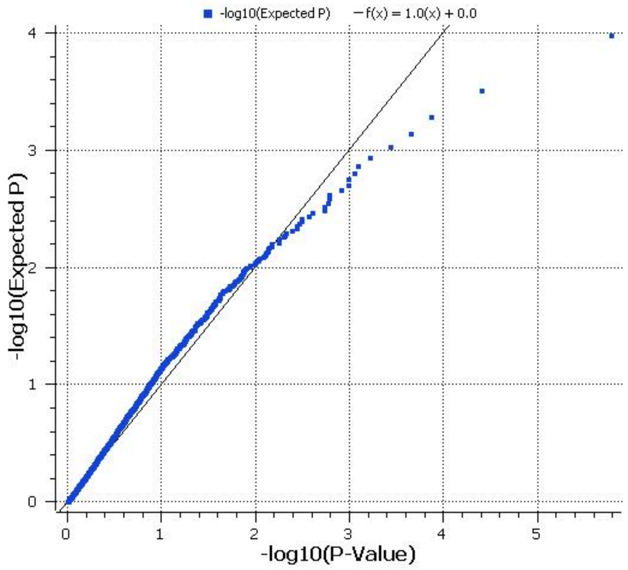

Leucine

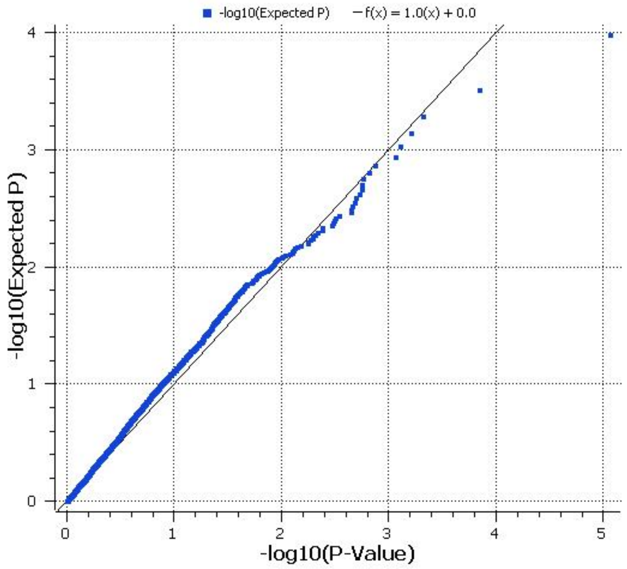

Phenylalanine

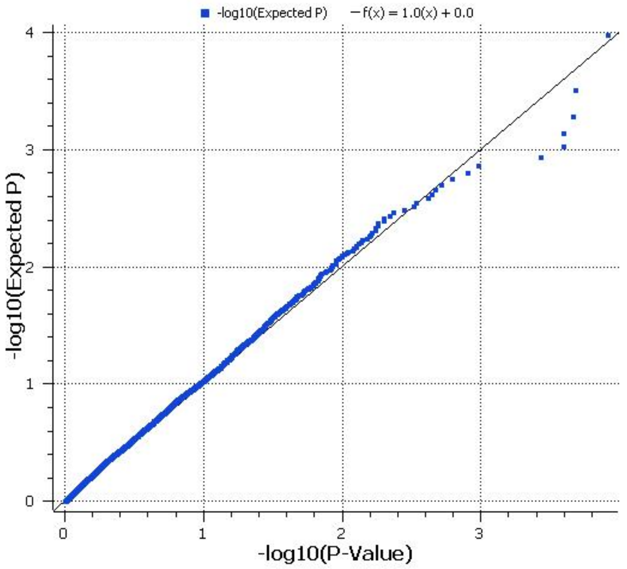

Ethanolamine

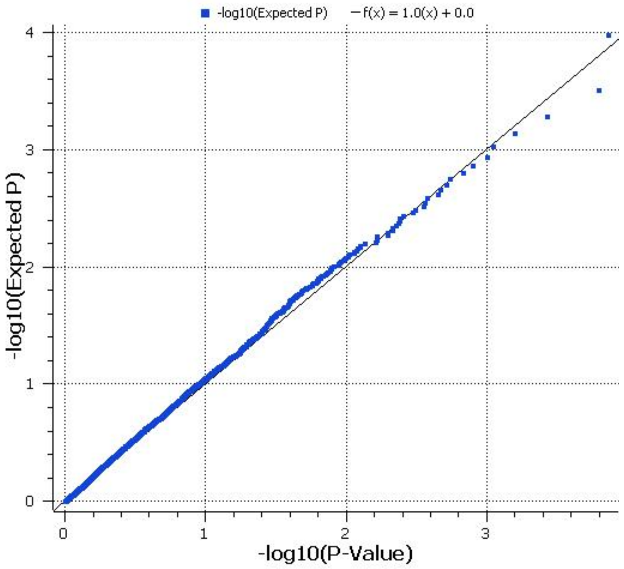

Hydroxylysine

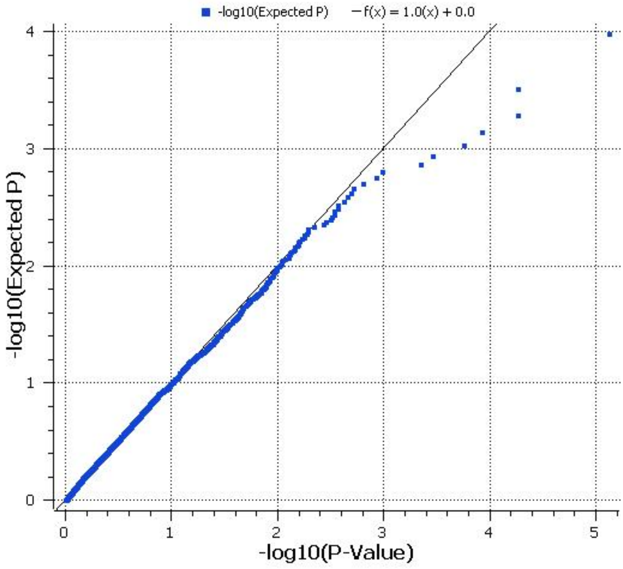

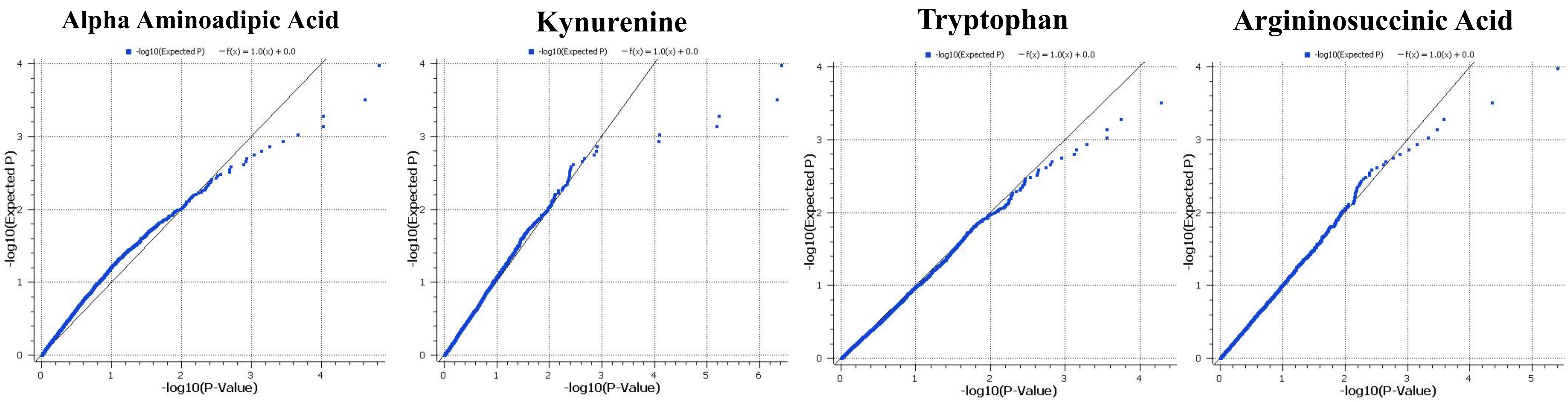

Supplemental Figure S7. Q-Q (quantile-quantile) plots of different amino acids. The plots were drawn for expected vs. observed -log10 (p-values) for all amino acids.
