# Supplementary material for: Genome-wide association study and population structure analysis of seed-bound amino acids and total protein in watermelon
Source: PeerJ. 2021 Oct 19;9:e12343. doi: 10.7717/peerj.12343 (PMC8533027; doi:10.7717/peerj.12343)
Supplement: Supplemental Information 8 — The plots was drawn for expected vs. observed −log10 (p-values) for total seed proteins [file peerj-09-12343-s008.pdf]

Total Proteins

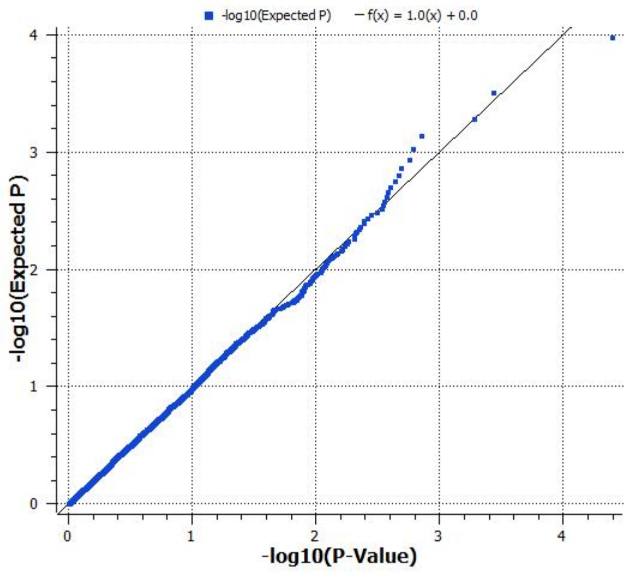

Supplemental Figure S8. Q-Q (quantile-quantile) plots of total proteins. The plots was drawn for expected vs. observed -log10 (p-values) for total seed proteins.
