# Supplementary material for: Genome-wide association study and population structure analysis of seed-bound amino acids and total protein in watermelon
Source: PeerJ. 2021 Oct 19;9:e12343. doi: 10.7717/peerj.12343 (PMC8533027; doi:10.7717/peerj.12343)
Supplement: Supplemental Information 9 [file peerj-09-12343-s009.pdf]

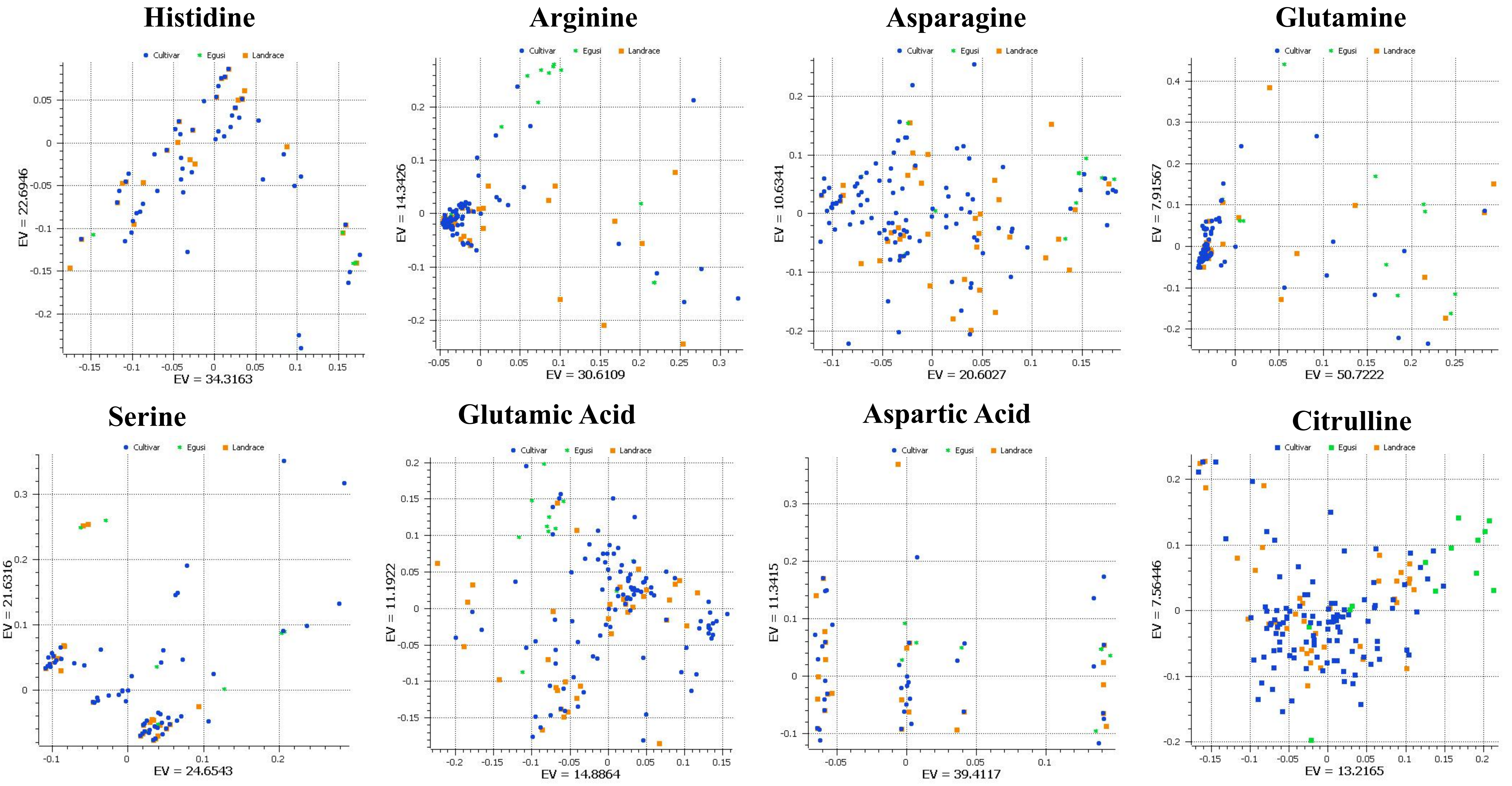

Supplemental Figure S9. Principal component analysis (PCA) for amino acids showing the components of population genetic variation

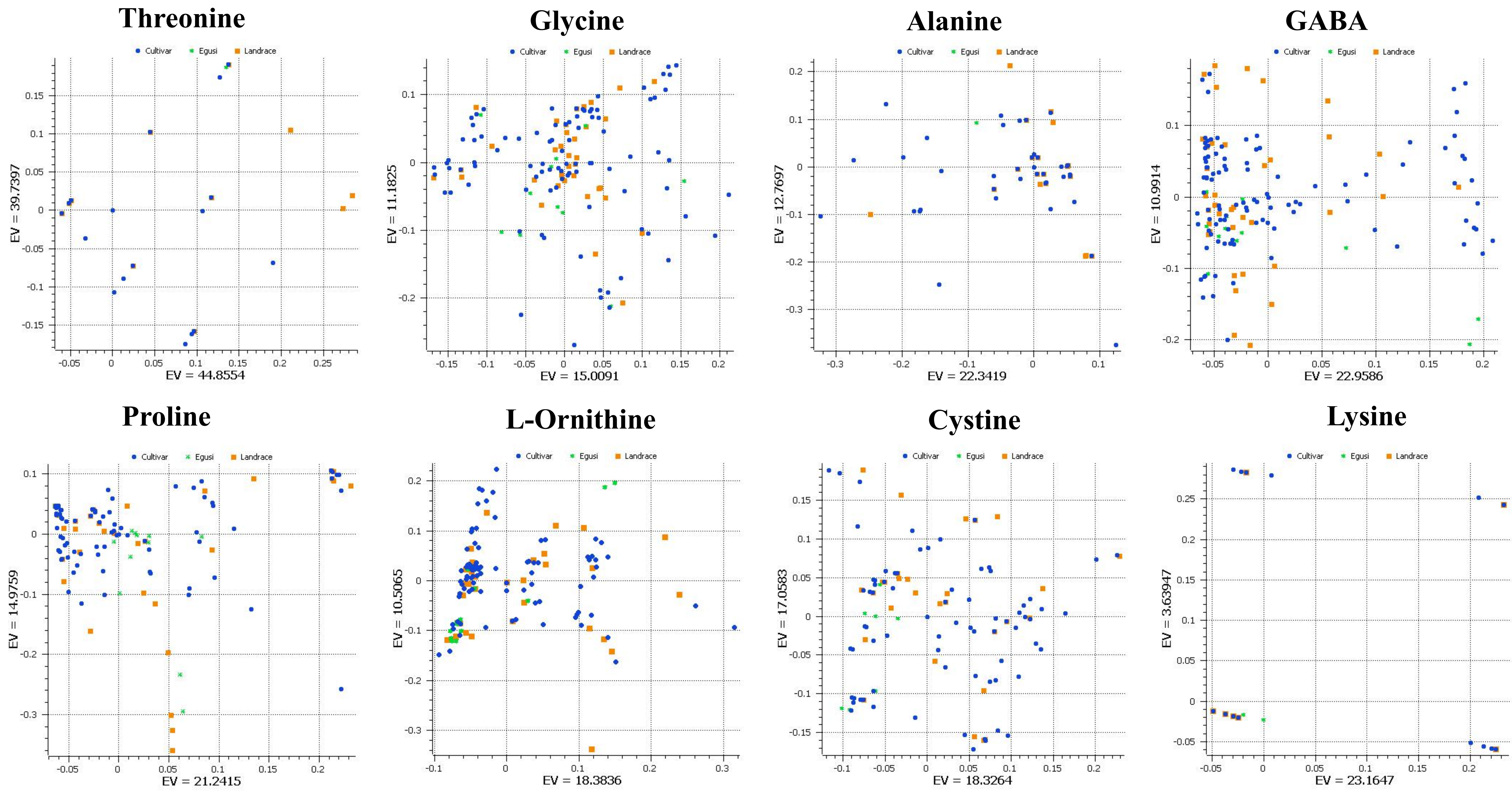

Supplemental Figure S9. Principal component analysis (PCA) for amino acids showing the components of population genetic variation

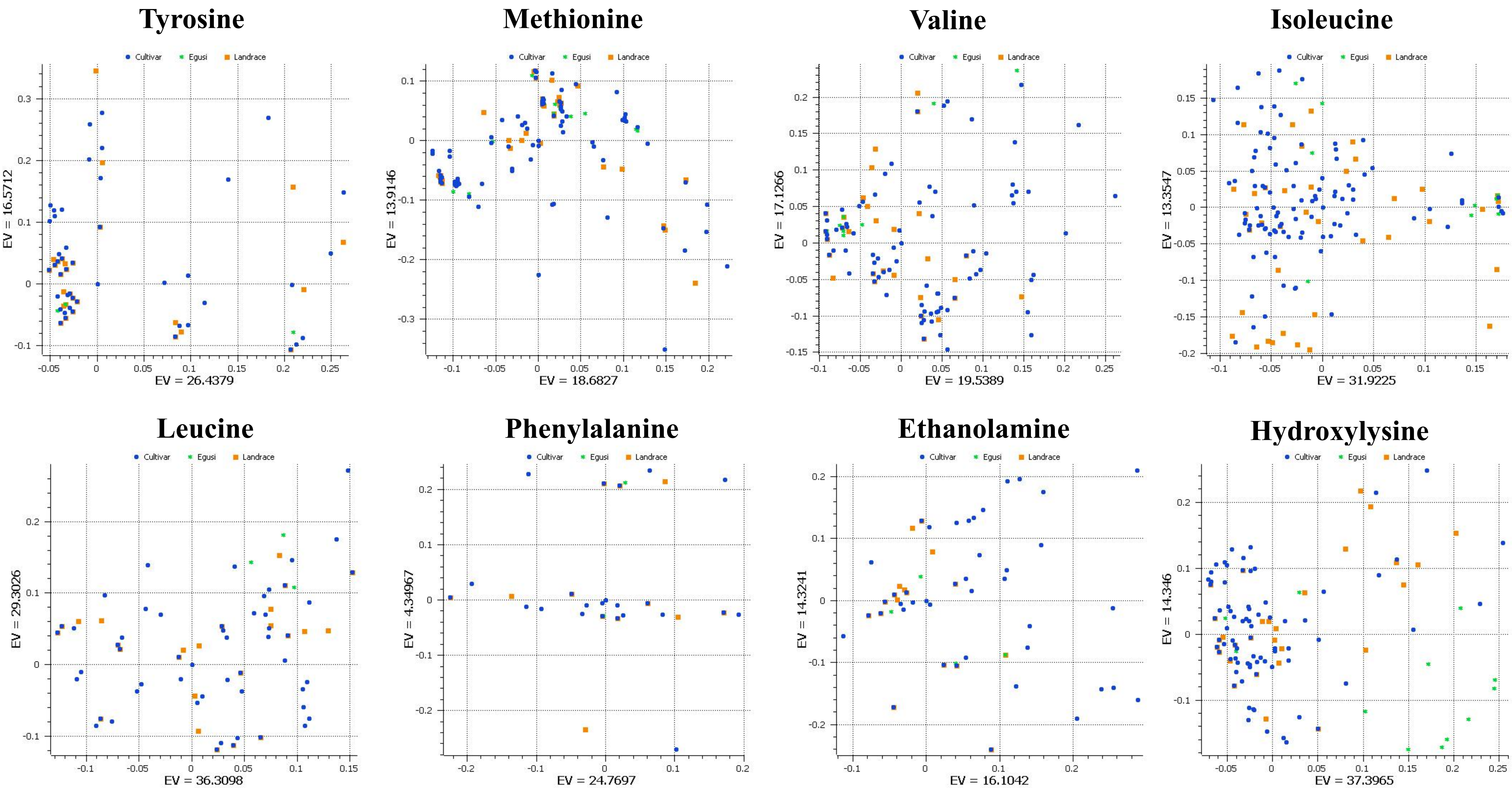

Supplemental Figure S9. Principal component analysis (PCA) for amino acids showing the components of population genetic variation

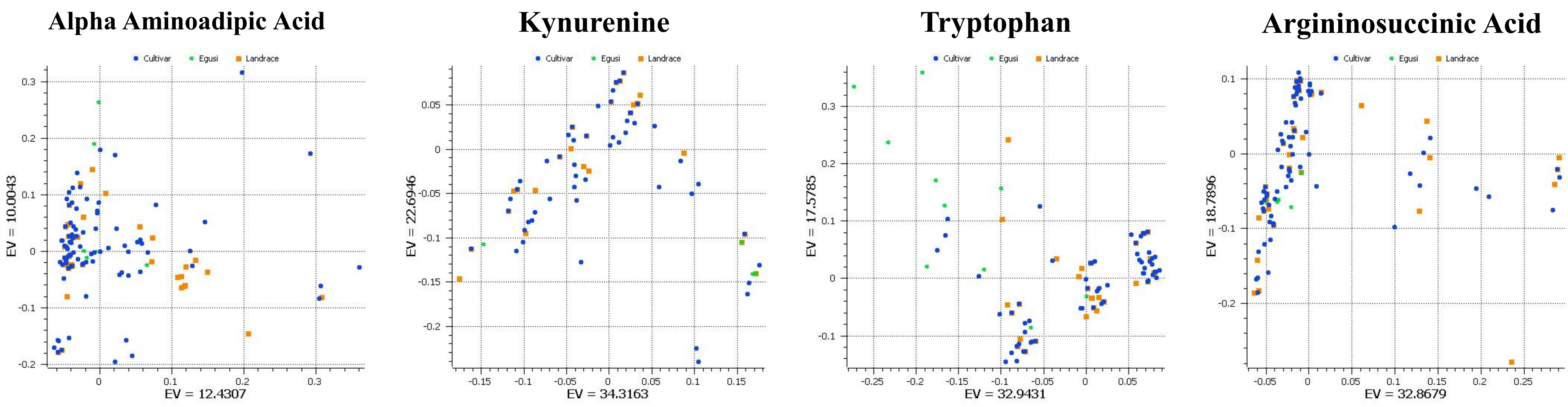

Supplemental Figure S9. Principal component analysis (PCA) for amino acids showing the components of population genetic variation
