# Supplementary material for: Genome-wide association study and population structure analysis of seed-bound amino acids and total protein in watermelon
Source: PeerJ. 2021 Oct 19;9:e12343. doi: 10.7717/peerj.12343 (PMC8533027; doi:10.7717/peerj.12343)
Supplement: Supplemental Information 10 [file peerj-09-12343-s010.pdf]

Total Proteins

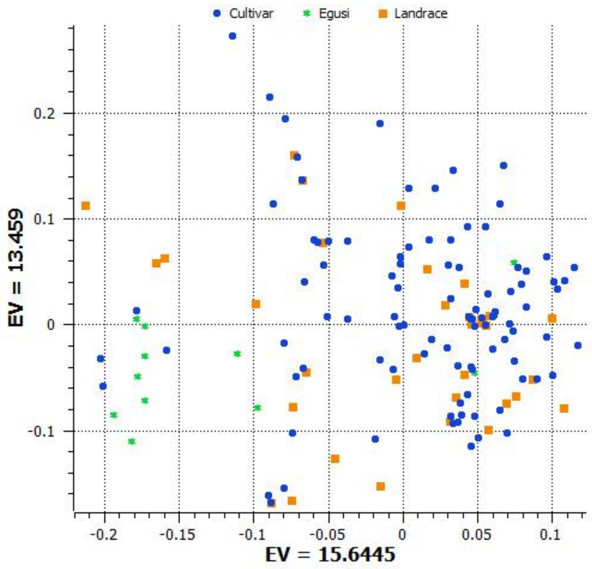

Supplemental Figure S10. Principal component analysis (PCA) of total proteins showing the components of population genetic variation
